# An advanced TSMK-FVC approach combined with Landsat 5/8 imagery for assessing the long-term effects of terrain and climate on vegetation growth

**DOI:** 10.3389/fpls.2024.1363690

**Published:** 2024-07-18

**Authors:** Zhenxian Xu, Xin Shen, Sang Ge, Qinglei Sun, Ying Yang, Lin Cao

**Affiliations:** ^1^ Co-Innovation Center for Sustainable Forestry in Southern China, Nanjing Forestry University, Nanjing, Jiangsu, China; ^2^ Yunnan Baima Snow Mountain National Nature Reserve Management Bureau, Shangri-La, Yunnan, China

**Keywords:** Landsat time series, fractional vegetation cover, spatio-temporal change of vegetation, topographic factors, climatic factors

## Abstract

**Introduction:**

As an exceptional geographical entity, the vegetation of the Qinghai-Tibetan Plateau (QTP) exhibits high sensitivity to climate change. The Baima Snow Mountain National Nature Reserve (BNNR) is located in the south-eastern sector of the QTP, serving as a transition area from sub-tropical evergreen broadleaf forest to high-mountain vegetation. However, there has been limited exploration into predicting the temporal and spatial variability of vegetation cover using anti-interference methods to address outliers in long-term historical data. Additionally, the correlation between these variables and environmental factors in natural forests with complex terrain has rarely been analyzed.

**Methods:**

This study has developed an advanced approach based on TS (Theil-Sen slope estimator) MK (Mann-Kendall test)-FVC (fractional vegetation cover) to accurately evaluate and predict the time and spatial shifts in FVC within the BNNR, utilizing the GEE (Google Earth Engine). The satellite data utilized in this paper consisted of Landsat images spanning from 1986 to2020. By integrating TS and MK methodologies to monitor and assess the FVC trend, the Hurst index was employed to forecast FVC. Furthermore, the association between FVC and topographic factors was evaluated, the partial correlation between FVC and climatic influences was analyzed at the pixel level (30×30m).

**Results and discussion:**

Here are the results of this research: (1) Overall, the FVC of the BNNR exhibits a growth trend, with the mean FVC value increasing from 59.40% in 1986 to 68.67% in 2020. (2) The results based on the TS-MK algorithm showed that the percentage of the area of the study area with an increasing and decreasing trend was 59.03% (significant increase of 28.04%) and 22.13% (significant decrease of 6.42%), respectively. The coupling of the Hurst exponent with the Theil-Sen slope estimator suggests that the majority of regions within the BNNR are projected to sustain an upward trend in FVC in the future. (3) Overlaying the outcomes of TS-MK with the terrain factors revealed that the FVC changes were notably influenced by elevation. The partial correlation analysis between climate factors and vegetation changes indicated that temperature exerts a significant influence on vegetation cover, demonstrating a high spatial correlation.

## 1 Introduction

Vegetation is an indispensable portion of land ecosystems. It has a critical function in carbon cycling, climate regulation and the maintenance of ecosystem sustainability at both regional and global scales (Duo et al., 2016; Fu et al., 2022b; Li et al., 2023). Nevertheless, there are distinct regional variations in the vegetation response to climate change, influenced by factors such as topography and geomorphology (Palombo et al., 2014; Guo et al., 2016). In environmentally sensitive regions, the impacts of climate change on vegetation phenology are likely to be amplified, especially in mountainous regions situated in geographic transition zones and at high altitudes (Nemani et al., 2003; Hua et al., 2017). National nature reserves are typically situated in mountainous regions characterized by abundant vegetation cover, serving as natural buffer zones that mitigate the impacts of climate variability and natural disasters (Xu et al., 2022). Therefore, amidst global climate change, it is imperative to effectively monitor vegetation within natural reserve areas in order to study how plant phenology responds to fluctuations in climate (Roberts et al., 2020; Zhang et al., 2021).

Earlier studies have utilized NDVI to track the vegetation cover (Zhang et al., 2018). However, NDVI itself has the limitation that it is easy to saturate in high-vegetation cover regions and is difficult to identify the tree canopies in low vegetation cover regions. Nevertheless, these issues can be addressed by calculating the fractional vegetation cover (FVC) (Ma et al., 2021). FVC represents the proportion of the vertically projected surfaces of plant stems, leaves, etc., to the total area within a given region. It can be utilized to track the development of vegetation cover (Marsett et al., 2006; Lehnert et al., 2015). Therefore, FVC can serve as an effective indicator of the vegetation assessment, reflecting the dynamic changes of vegetation affected by various elements such as climate shift, land cover variation, and environmental projects (Geng et al., 2022; Han et al., 2023). On the other hand, long time series-based FVC allows for the analysis of vegetation cover changes in the study area and require the integration of multiple change monitoring methods (Zhu et al., 2021; Fu et al., 2023). Currently, several change detection algorithms have been applied to vegetation monitoring, such as LandTrendr algorithm (Kennedy et al., 2010), continuous change detection and classification (CCDC) algorithm (Zhu and Woodcock, 2014) and BFAST algorithm (Verbesselt et al., 2010). However, commonly used methods of FVC trend analysis include regression analysis and TS (Theil-Sen slope estimator)-MK (Mann-Kendall analysis) analysis (Liu et al., 2022; Fu et al., 2022b). The advantage of TS-MK is that the data do not need to obey a certain distribution law, the data error has a strong resistance to the data, for the significance level test has a more reliable statistical theory basis, so that the results of the operation are more scientific and credible (Zhang et al., 2022b). In addition, Hurst index is widely used in studies of the future sustainability of vegetation cover (Zhang and Jin, 2021; Zhu et al., 2021). Coupling the Hurst exponent with the Theil-Sen slope estimator allows for a more accurate representation of future trends in vegetation. Therefore, this paper integrates the Mann-Kendall, Theil-Sen slope and Hurst index methods to analyze the trend, significance, and future projection of vegetation changes.

Field measurements and remote sensing techniques are available for monitoring the spatial and temporal dynamics of vegetation. However, field measurements encounter limitations such as the inability to gather continuous observation data over extended periods, high monitoring costs and difficulties in realizing large-area monitoring (Han et al., 2023). Remote sensing technology has emerged as a promising approach for monitoring vegetation growth, owing to its ability to provide continuous spatial coverage and long-term data series (Chu et al., 2019; Li and Yang, 2023). Normally, long time-series remote sensing imagery and its derived products can be used to estimate FVC that is beneficial for monitoring the vegetation changes in large river basins, urban agglomerations, and grasslands (Xiao et al., 2017; Geng et al., 2022). Traditional remote sensing analysis methodologies require downloading and pre-processing a large number of images. Google Earth Engine (GEE) has already carried out atmospheric correction, geometric registration, radiometric calibration and other pre-processing of commonly used images. It enables swift realization of image acquisition, batch processing, calculation and analysis by means of on-line programming, which can greatly improve the operational efficiency (Hansen et al., 2013; Gorelick et al., 2017). With the GEE cloud platform, the FVC of the study area can be uniformly and quickly estimated, and the long time series dynamic monitoring of vegetation can be realized (Fu et al., 2022b).

Geographic conditions at high altitudes are relatively complex, with strong spatial heterogeneity in vegetation cover and climate. Therefore, the influence of topographic and climatic factors on highland vegetation has received widespread attention (Wei et al., 2022; Han et al., 2023; Xu and Wu, 2023). At the same time, compared with low-altitude areas, high-altitude areas are often different from each other and cannot be generalized. Even within the same geographic unit, there are differences in the response of vegetation to climatic factors such as temperature and precipitation (Deng et al., 2022b). However, current vegetation monitoring at high altitudes tends to focus on the entire plateau area, using coarse resolution (>500m) AVHRR or MODIS remote sensing imagery to analyze spatial and temporal changes in vegetation (Zhang and Jin, 2021; Han et al., 2023; Huang et al., 2023). It was shown that the spatial resolution of remote sensing images may affect the accuracy of FVC estimation, and suitable remote sensing images need to be selected according to the study area (Zhang et al., 2014; Wang et al., 2022). Landsat imagery has not only higher spatial resolution but also longer time series, which is more suitable for monitoring vegetation at high altitudes at medium regional scales (Wang et al., 2022). Few studies have focused on the investigation of coverage changes of vegetation in mountainous areas. Moreover, the reactions of vegetation to climate variation and topography in complex terrain regions were seldom analyzed. The Baima Snow Mountain National Nature Reserve (BNNR) is located in the transition zone between the Tibetan Plateau and the Yunnan-Guizhou Plateau. The terrain is complex and diverse, with large elevation differences (>3500 m) and a rich variety of vegetation types (Ning et al., 2012). This geomorphological feature makes the vegetation in the BNNR more sensitive to topographic and climatic factors. Therefore, the BNNR is an ideal site for studying the effects of climate change on vegetation growth. By conducting research in the area, it is possible to better understand how topographic and climatic factors interact, providing valuable references and recommendations for ecological conservation. Therefore, in order to realize the monitoring variability of vegetative cover in nature reserves and find relationships of the variations to terrain and climate shifts, the purposes of this research involve: (1) to quantitatively estimate of long time series FVC (1986-2020) in the whole study area with complex terrain, and analyze the spatial and temporal change of vegetative cover; (2) to develop a TS (Theil-Sen slope estimator) MK (Mann-Kendall test) - FVC based approach for accurately evaluating and predicting of the spatio-temporal changes of vegetative coverage, and investigate the reaction of vegetative cover to topography and climate in the BNNR.

## 2 Materials

### 2.1 Study area

**Figure 1:**
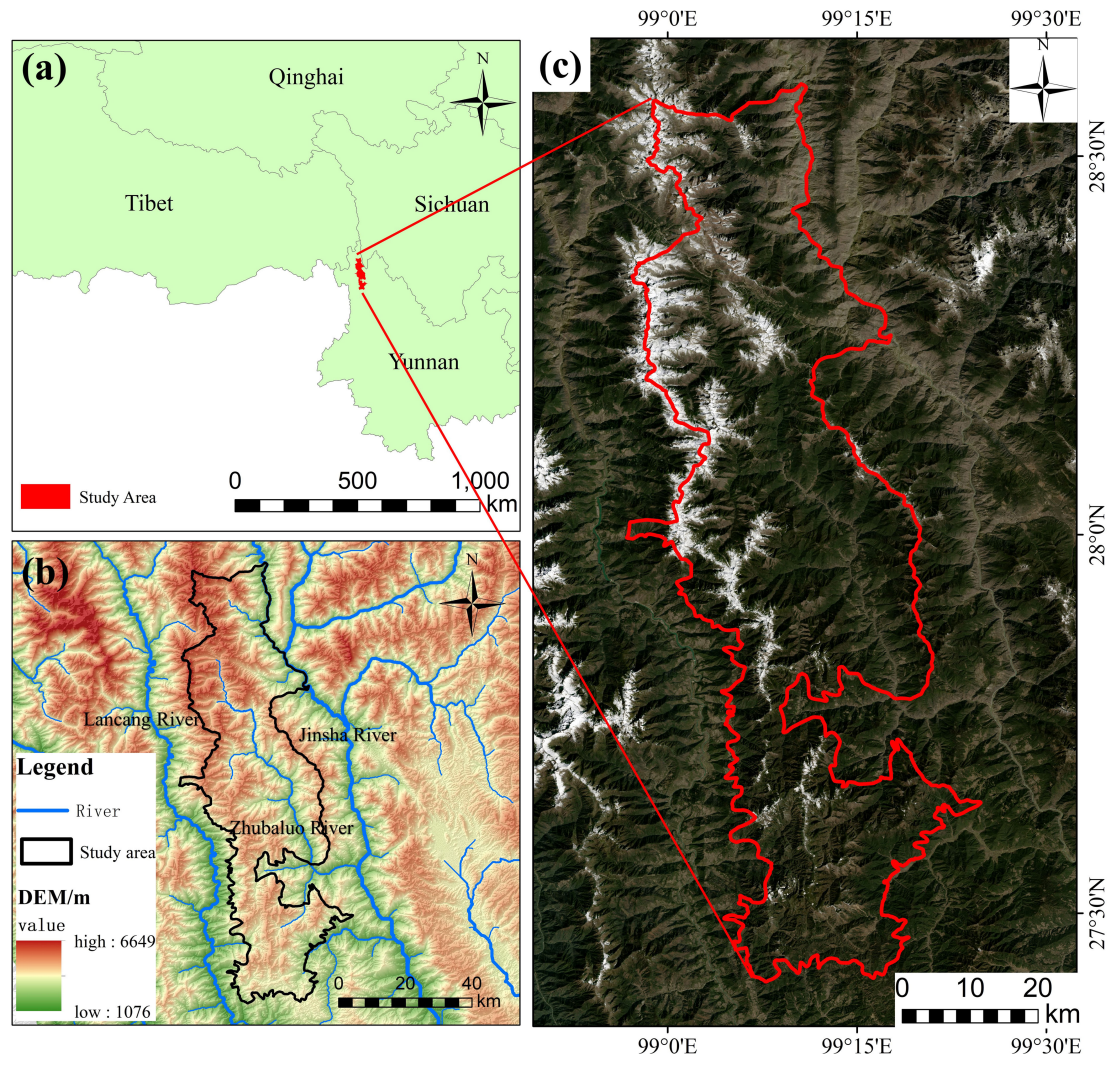
Overview of Baima Snow Mountain National Nature Reserve (BNNR); **(A)** location of the BNNR; **(B)** topography; **(C)** Landsat image (2020).

The Baima Snow Mountain National Nature Reserve is situated in Diqing Tibetan Autonomous Prefecture, Yunnan Province. Its northern portion belongs to Deqin County, while its southern part lies within Weixi County. The geographical coordinates of the BNNR are between 98°57′-99°25′E and 27°24′-28°36′N. Within the core of the Hengduan Mountains is where the BNNR is situated, between the Jinsha River and the Lancang River, the famous Three Parallel Rivers Region. The main river flowing through the reserve is the Zhubaluo River, which originates from the Baima Snow Mountain, with a drainage area of 1835km^2^. The BNNR has a prominent monsoon climate characterized by distinct wet and dry seasons, and rainfall is mainly centered in July and August. Furthermore, the BNNR’s precipitation distribution is spatially inhomogeneous. Due to topographic and climatic factors, precipitation is much higher in the valley of the Zhubaluo River and the south than in other areas. The BNNR is rich in biodiversity and is an important habitat for the worldwide precious and threatened species and national-level protected animal, the Yunnan snub-nosed monkey. The perpendicular arrangement of vegetation in the region is obvious. Perpendicular distribution of vegetation can be separated into river valley shrubs and tussock, broadleaf forest, coniferous forest, and meadow according to the altitude from 2500m to 5000m (Sun et al., 2017; Su et al., 2022). Examining the features of the vegetation’s temporal and spatial distribution as well as the impacts of topography and climate on plants is crucial given the unique topographic and climatic circumstances of the BNNR (see **Figure 1**).

### 2.2 Remote sensing data

Satellite images utilized in this study include Landsat 5 (1986-2012) and Landsat 8 (2013-2020). The images were acquired from the USGS. Landsat Surface Reflectance (SR) data for the years 1986–2020 were obtained using the Google Earth Engine (GEE) platform (https://code.earthengine.google.com/). The spatial resolution of the data is 30m, and it has been pre-processed including atmospheric correction and geometric correction. Since most areas of the BNNR were covered by clouds all year round, the remote sensing images need to be de-clouded. The QA quality band of SR remote sensing images was operated bit by bit to realize the filtering of pixel values. Masking of clouds, cloud shadows and snow pixels of remote sensing images to finally remove clouds and snow (Zhu and Woodcock, 2012).

**Figure 2:**
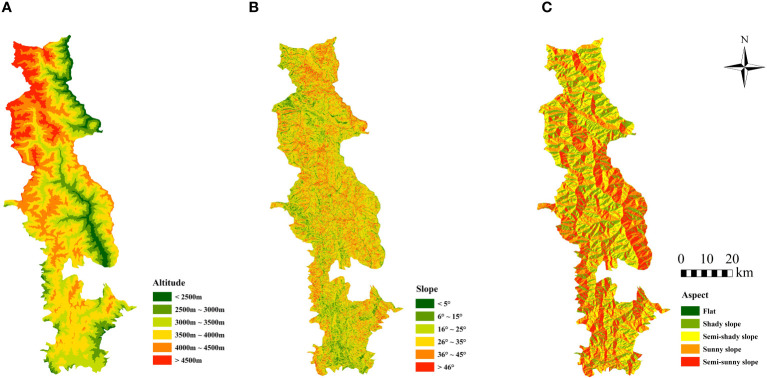
Spatial distribution of terrain factor classification results in BNNR; **(A)** altitude was divided into 6 categories; **(B)** slope was categorized into 6 categories; **(C)** aspect is categorized into 5 categories.

By processing SRTM DEM data, derived data products including altitude, slope, and aspect are generated. The spatial resolution of the DEM data is 30 m. Based on the local conditions of the BNNR and related literature (Wang et al., 2021b; Deng et al., 2022a), the topographic data were reclassified (**Figure 2**). In order to examine the influence of terrain on vegetative cover, FVC computations will be spatially overlaid with altitude, slope, and aspect data in subsequent analyses.

Meteorological data can be downloaded from the National Earth System Science Data Center website (http://www.geodata.cn/). The dataset includes the 1 km resolution monthly precipitation and average temperature dataset between 1986 and 2020. This data was cropped, resampled and algebraically manipulated to finally obtain the temperature and precipitation data of BNNR (Peng et al., 2019).

Data on vegetative cover categories were downloaded from Data Center for Environmental and Resources Sciences (https://www.resdc.cn/). The dataset comprises 11 vegetation categories, of which 7 are distributed within BNNR.

## 3 Methods

### 3.1 The long time series FVC calculation based dimidiate pixel model

**Figure 3:**
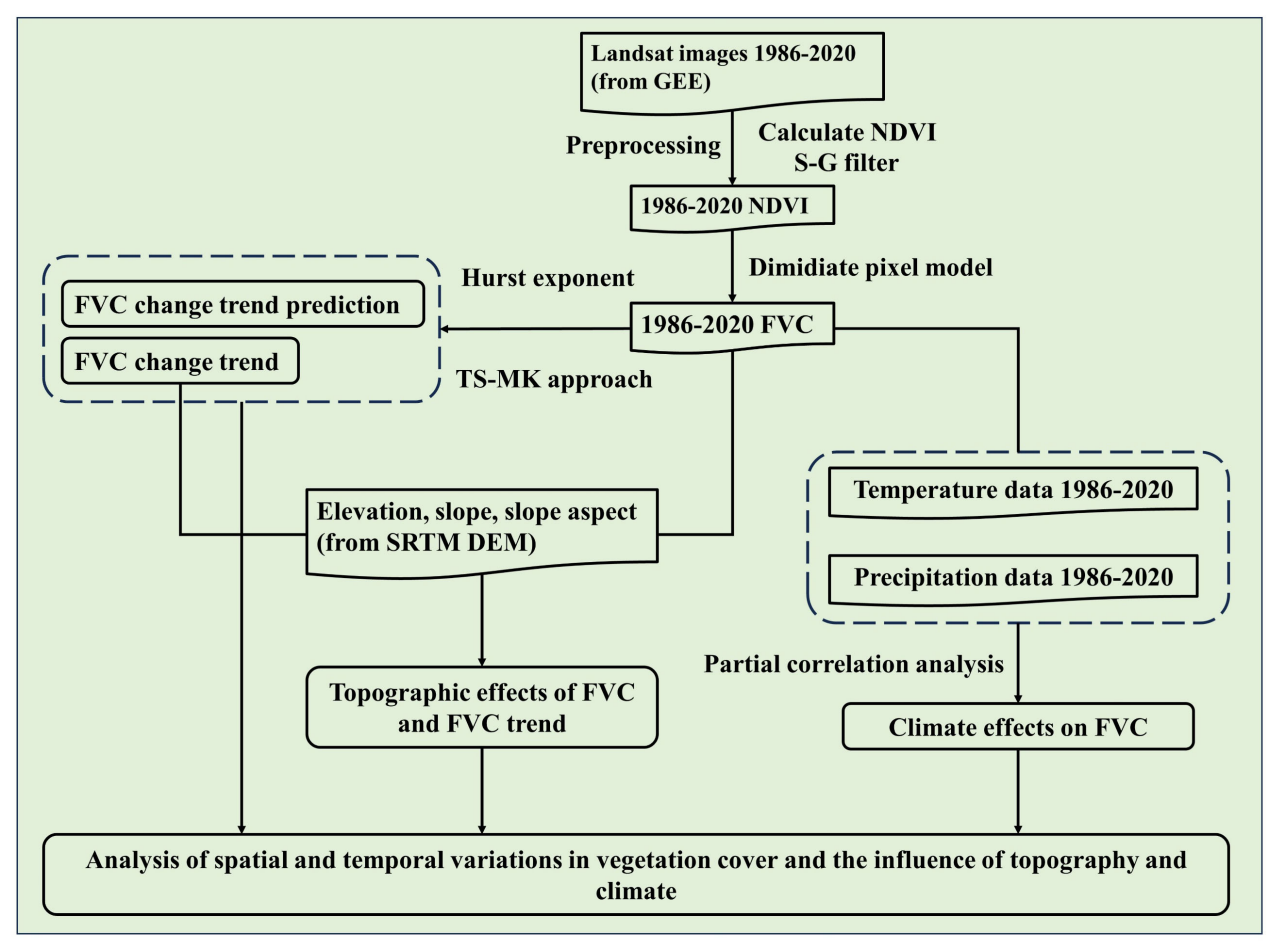
Flow chart of assessing and predicting the spatio-temporal variability of vegetation cover based on an advanced TSMK-FVC approach using Google Earth Engine in BNNR.

NDVI is normally seen as an effective index reflecting large-scale vegetation coverage and growth status, and is among the most extensively applied vegetation indices (Schultz et al., 2016; Lu et al., 2019; Vulova et al., 2023). In this research, Surface Reflectance (SR) images of Landsat were used to calculate NDVI in accordance with GEE cloud platform, and Maximum Value Composites (MVC) were applied to composite the NDVI data spanning from 1985 and 2020 (Lanorte et al., 2014; Liu et al., 2022). Owing to the effects of satellite sensor performance, cloud cover and atmospheric conditions, NDVI time series dataset has serious noise (Goward et al., 1991). Therefore, it was imperative to correctly and effectively remove noise and reconstruct NDVI time series dataset before application. Previous research has demonstrated that S-G filtering can effectively improve the data quality of vegetation index products (Chen et al., 2004; Shao et al., 2016). In this paper, S-G filtering method was for application in rebuild time series of NDVI data to remove noise (see **Figure 3**) (Zhao and Zhang, 2018; Han et al., 2020).

Further calculation of FVC based on NDVI can help alleviate the issue of NDVI saturation in monitoring areas with high vegetation cover and difficulty in identification in areas with low vegetation cover. In many studies, there were three typical methods to estimate vegetation cover using remote sensing data, such as the Empirical method, the Spectral Mixture Analysis method and the DPM (Jiapaer et al., 2011). The DPM is a fast and effective method for calculating FVC because it is easy to operate and has performed well (Shi et al., 2021b; Hill and Guerschman, 2022). The principle of the DPM involves assuming that the reflectance *(R)* of a pixel consists of two components: the part with vegetation cover *(R_V_)* and the part without vegetation cover *(R_S_)*. Subsequently, the spectral information observed through the sensor is synthesized by the linear weighting of these two components.


(1) $$R=R_{V}+R_{s}$$

The proportion of the area of a pixel that has vegetation cover is FVC, which is the vegetation cover percentage of the pixel. Consequently, the proportion of the area that is not covered by vegetation is *(1-FVC)*. The reflectance of a pixel is *R_veg_* if the pixel is completely covered by vegetation and *R_soil_* if it is completely covered by soil. The information contributed by the vegetated portion of the hybrid pixel can be expressed as the product of the purely vegetated reflectance, *R_veg_*, and the area covered by vegetation in the image element, *FVC*. And the information contributed by the non-vegetated component can be expressed as the product of *R_soil_* and *(1-FVC)*.


(2) $$R_{V}=R_{veɡ}\times FVC$$

(3) $$R_{s}=R_{soil}\times \left(1-FVC\right)$$

By solving Equations 1–3, the FVC can be calculated as Equation 4:


(4) $$FVC=\frac{R-R_{soil}}{R_{veɡ}+R_{soil}}$$

In the inversion of FVC using NDVI, NDVI can be used instead of *R*. This results in the formula for calculating FVC based on NDVI (see Equation 5):


(5) $$FVC=\frac{NDVI-NDVI_{soil}}{NDVI_{veɡ}+NDVI_{soil}}$$

Since the theoretical values of bare ground *NDVI_soil_* and pure vegetation cover *NDVI_veg_* should be close to 0 and 1, respectively. The FVC was calculated by intercepting the upper and lower NDVI thresholds with 5% confidence, and averaging the 5% areas with the smallest and the largest NDVI values, respectively, to obtain the *NDVI_soil_* and *NDVI_veg_*.

### 3.2 FVC dynamic analysis method

#### 3.2.1 Trend in FVC

With the objective of investigating the spatial distribution and temporal variations of vegetative cover in the BNNR from 1986 to 2020. Tendency and stability characteristics of vegetation cover have been investigated utilizing the Mann-Kendall test (MK) and the Theil-Sen slope estimator (TS).

TS is considered suitable for investigating the slope tendency in time series data (Sun et al., 2021). This method has advantages including insensitivity to measurement error and group data as well as high computational efficiency (Peng et al., 2015). MK is one of the commonly used tools for nonparametric trend testing, which is not necessary that the data samples follow a specific pattern, as well as can be used to test the trend of data changes under long time series (Yue et al., 2002; Luo et al., 2020). Combining TS and MK will provide a strong anti-interference ability for data noise, and the specific distribution of analysis data is not a prerequisite (Zhang et al., 2022b). Thus, TS-MK was applied to investigate the trend of the dynamic shifts in FVC of the BNNR. The formula of TS equation is as follows (see Equation 6):


(6) $$S_{FVC}=median\frac{FVC_{b}-FVC_{a}}{b-a}$$

Where 1< *a*< *b< n*, *a* and *b* represent the amounts of years in the time series. *FVC_b_* and *FVC_a_* are the FVC values of time series *a* and *b*, respectively. When the slope *S_FVC_* is greater than 0, it indicates *a* growth tendency; when it is less than 0, it indicates a downward tendency.

The calculation formula of Manna-Kendall test is shown in Equations 7–10:


(7) $$\text{S}=\sum_{j}^{n-1}\sum_{i=j+1}^{n}sɡn\left(FVC_{j}-FVC_{i}\right)$$

(8) $$sɡn\left(FVC_{j}-FVC_{i}\right)=\{\begin{array}{cc} 1 & \;FVC_{j}-FVC_{i}>0\\ 0 & \;FVC_{j}-FVC_{i}=0\\ -1 & \;FVC_{j}-FVC_{i}<0 \end{array}\;$$

(9) $$\text{Z}=\{\begin{array}{cc} \frac{\text{S}-1}{\sqrt{\mathrm{Var}\left(\text{S}\right)}} & \left(S>0\right)\\ 0 & \left(S=0\right)\\ \frac{S+1}{\sqrt{\text{Var}\left(S\right)}} & \left(S<0\right) \end{array}$$

(10) $$\text{Var}\left(S\right)=\frac{n\left(n-1\right)\left(2n+5\right)}{18}$$

Where *S* is the correlation coefficient of the Mann-Kendall test; *Z* is the significance index, and its value range is (-∞, +∞), which follows the standard normal distribution. *Z*-value greater than 0 represents a significant increasing tendency, while a *Z*-value less than 0 represents a significant decreasing trend.

#### 3.2.2 FVC trend prediction

The Hurst exponent was employed in this research to forecast the FVC trend in the BNNR going forward. Hurst originally introduced the Hurst exponent, which is a way to determine if continuous time series data exhibit long-term correlation (Bashir et al., 2020). This exponent was established using R/S analysis on the basis of long-term hydrological observations (Zhang and Jin, 2021). The Hurst index is generally in the range from 0 to 1. When 0< *H*< 0.5, the future tendency of FVC will be reversed; when *H*=0.5, the development trend of FVC is unpredictable; when *H* is greater than 0.5 and less than 1, the future time series of FVC remains in agreement with what has happened previously (Tong et al., 2018; Liu et al., 2022). The selection of the time window for the Hurst exponent requires consideration of the study object and the cloudiness of the study area (Zhu et al., 2021; Fu et al., 2022b). Since the dominant tree species in the study area are all evergreen and have high cloudiness in the growing season. Therefore, to achieve year-by-year time series FVC estimation in the study area, FVC with a time window of the non-growing season was used for Hurst exponent calculation. The Hurst exponent is calculated as shown in Equations 11–15.

Define the FVC time series as *FVC(τ)*, The mean of this time series is:


(11) $$\bar{{\text{FVC}}_{\left(\text{τ}\right)}}=\frac{1}{\text{τ}}\sum_{\text{t}=1}^{\text{τ}}{\text{FVC}}_{\left(\text{t}\right)},\text{τ}=1,2,3\cdots ,\text{n}$$

The cumulative deviation is:


(12) $$U\left(t,\tau \right)=\sum_{t=1}^{\tau }(\text{FV}C_{\left(t\right)}-\bar{\text{FV}C_{\left(\tau \right)}})\;,1\leq t\leq \tau$$

The range *R(τ)* is:


(13) $$R_{\left(\tau \right)}=\text{max}U_{\left(t,\tau \right)}-\text{min}U_{\left(t,\tau \right)}$$

The standard deviation sequence is:


(14) $$S_{\left(\tau \right)}=\sqrt{\frac{1}{\tau }\sum_{\tau =1}^{\tau }{\left(\text{FV}C_{\left(t\right)}-\bar{\text{FV}C_{\left(\tau \right)}}\right)}^{2}}$$

Calculate the Hurst exponent:


(15) $$R_{\left(\tau \right)}/S_{\left(\tau \right)}={\left(\alpha \tau \right)}^{H}$$

where *H* represents the Hurst exponent in the range greater than 0 and less than 1.

Afterwards, the Hurst exponent and TS results were overlaid to get coupled data on the changes’ tendency and consistency, and the results were classified as: (1) decrease to increase; (2) consistent decrease; (3) increase to decrease; (4) consistent increase (see **Table 1**).

**Table 1:** Future trend categories of FVC combining Hurst exponent and TS.

Continuation/FVC trends	FVC decrease trend	FVC increase trend
Consistent trends (0.5< *H*< 1)	Consistent decrease	Consistent increase
Inconsistent trends (0< *H*< 0.5)	Decrease to increase	Increase to decrease

### 3.3 Relevance analysis of FVC with climatic elements

Pixel-by-pixel calculation of the relevance among FVC data and climatic elements for the BNNR from 1986 to 2020. The correlation coefficients were calculated using the Equation 16:


(16) $$R_{ab}=\frac{\sum_{i=1}^{n}\left(x_{i}-\bar{x}\right)\left(y_{i}-\bar{y}\right)}{\sqrt{\sum_{i=1}^{n}{\left(x_{i}-\bar{x}\right)}^{2}}\sqrt{\sum_{i=1}^{n}{\left(y_{i}-\bar{y}\right)}^{2}}}$$

where *R_xy_* is the correlation coefficient of factor *x* and factor *y*.

When multiple factors are correlated with FVC simultaneously, the use of partial correlation analysis allows individual variables to be analyzed separately for their degree of correlation with FVC (Yuan et al., 2019). The equation of partial correlation analysis is shown below (see Equation 17):


(17) $$R_{xy,z}=\frac{R_{xy}-R_{xz}R_{yz}}{\sqrt{\left(1-R_{xz}^{2}\right)\left(1-R_{yz}^{2}\right)}}$$

Where *R_xy_,_z_* denotes the partial correlation coefficient of the *x* and *y* variables fixed factor *z* after. *R_xy_*, *R_xz_*, and *R_yz_* are the correlation coefficients of their variables, respectively. Where the values of the coefficients range from -1 to 1.

## 4 Results

### 4.1 Temporal and spatial properties of FVC

#### 4.1.1 Characterization of spatial distribution of FVC

**Figure 4:**
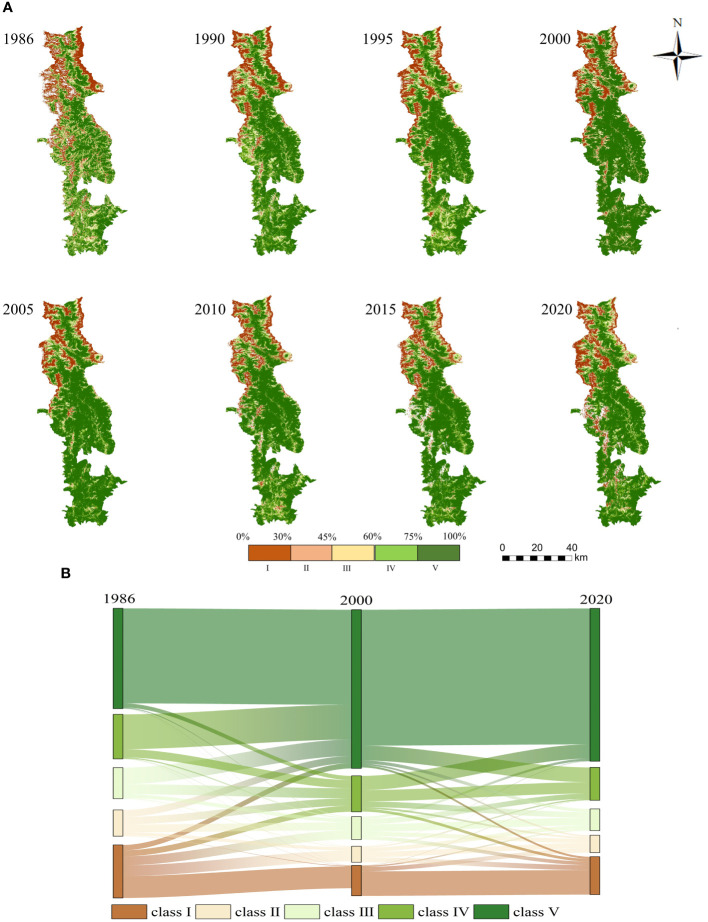
Modifications in the space distribution of FVC throughout the BNNR; **(A)** space distribution of FVC by class in the research region from 1986 to 2020; **(B)** sankey diagram of FVC proportion shift of different classes in the research region from 1986 to 2020.

According to the FVC classification standards and pertinent studies (Liu et al., 2022; He et al., 2023), FVC values were categorized into five classes and corresponded to different landscapes (see **Table 2**). **Figure 4A** illustrates the spatial distribution of FVC in the BNNR between 1986 and 2020. Areas of higher FVC are situated primarily at the BNNR’s eastern and southern regions. The northern and west-central snow-covered areas of the study area have lower FVC. Compared to 1986, the Class V FVC coverage area in 2000 and 2020 showed significant growth, primarily located in the BNNR’s central and southern regions.

**Table 2:** FVC is categorized into five categories and landscapes.

Vegetation coverage value	Categories	Landscape
FVC< 30%	class I	Bare land, snow-covered land, etc.
30% ≤ FVC< 45%	class II	Valley scrub, grassland, etc.
45% ≤ FVC< 60%	class III	Grassland, cropland, etc.
60% ≤ FVC< 75%	class IV	Shrubland, etc.
75%< FVC	class V	Woodland, etc.

#### 4.1.2 Characterization of FVC variation over time

**Figure 5:**
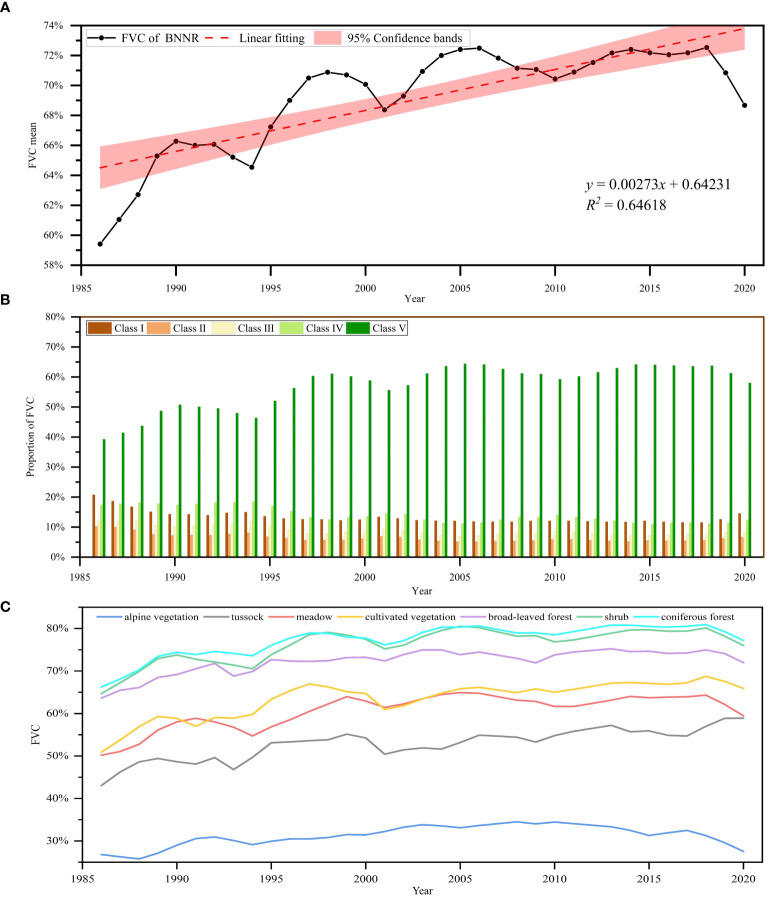
Temporal variation in FVC in the BNNR; **(A)** Interannual change in the mean FVC from 1986 to 2020; **(B)** changes in FVC by class in the BNNR from 1986 to 2020; **(C)** variations in mean FVC for various vegetation categories from 1986 to 2020.

The mean FVC value in the research region has exhibited a tendency to increase in a wavering manner over these thirty-five years (see **Figure 5A**). The average FVC increases from 59.40% in 1986 to 68.67% in 2020, an increase of 0.26% per decade. Throughout this 35-year period, the average annual FVC experienced two significant declines, with the turnaround occurring in 1994 and 2001. The decline in FVC may be attributed to climate-related disasters, and the implementation of ecological policies, such as the return of grazing land to forests, has to some extent contributed to the recovery of vegetation. The highest mean FVC of 72.53% occurred in 2018. The lowest mean FVC was 59.41% in 1986.

Further statistics were made on the changing trend of FVC of different classes in BNNR between 1986 and 2020. Among these classes, FVC for class I, II, III, and IV tended to decline, decreasing by 6.19%, 3.64%, 3.93%, and 5.00%, respectively. The corresponding class V showed an obvious upward trend, rising from 39.28% in 1986 to 58.03% (see **Figures 4B**, **5B**). The improving trend in fast FVC in the BNNR is dominated by the continued growth of high-class FVC.

A total of seven vegetation types (alpine vegetation, coniferous forest, broadleaf forest, meadow, shrub, tussock, and cultivated vegetation) were distributed in the BNNR. The more widely distributed vegetation types are coniferous forest and shrub, accounting for 32.51% and 31.79% of the BNNR, respectively. The FVC of all vegetation types exhibited an upward trend to varying degrees during these 35 years, with the more significant increases mainly in tussock (15.86% increase), cultivated vegetation (14.97% increase), shrub (11.32% increase), and coniferous forest (10.94% increase) (see **Figure 5C**).

### 4.2 FVC change trend and prediction

#### 4.2.1 FVC change trend

**Figure 6:**
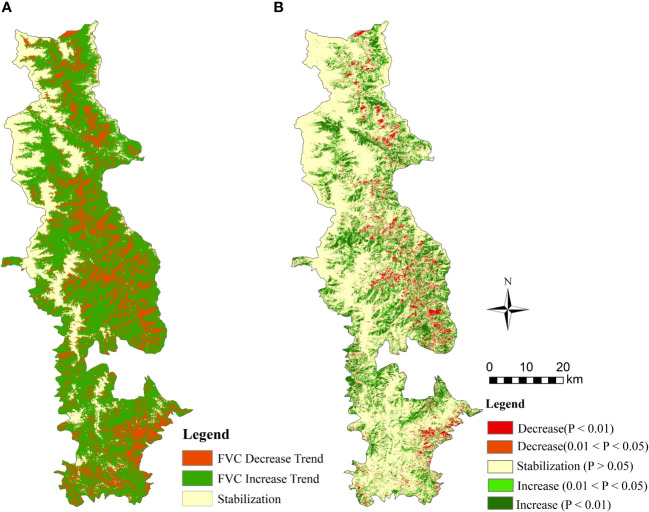
Spatial distribution of FVC 1986-2020 tendency in the BNNR; **(A)** the findings of TS analysis; **(B)** tendency in FVC 1986-2020 in the BNNR combining TS and MK.

The results of TS analysis can be classified into three categories based on the slope of FVC changes: *S_FVC_* < -0.0005, -0.0005< *S_FVC_* < 0.0005, and *S_FVC_* > 0.0005, indicating decreasing, no change, and increasing trends in FVC, respectively (see **Table 3**). As illustrated in **Figure 6A**, the area of the BNNR with an upward trend is 1651.57km^2^ and the area with a downward trend is 619.13km^2^, which account for 59.03% and 22.13% of the area, individually.

**Table 3:** TS (*S_FVC_*) and MK (Z-value) were combined to investigate the tendency of FVC and the findings were classified into five groups.

S_FVC_	Z-value	category	area/km^2^
*S_FVC_* < -0.0005	2.58< *|Z|*	Extremely significant decrease	109.38
	1.96< *|Z|* ≤ 2.58	Significant decrease	63.01
-0.0005< *S_FVC_* <0.0005	*|Z|*< 1.96	No significant change	1901.94
*S_FVC_* > 0.0005	1.96< *|Z|* ≤ 2.58	Significantly increase	284.53
	2.58< *|Z|*	Extremely significant increase	439.16

Among the area proportion of each trend, 61.48km^2^ and 118.23km^2^ of FVC were significantly reduced and very significantly decreased, accounting for 2.20% and 4.23% of the BNNR (see **Figure 6B**). The area where the decrease of FVC occurred is mainly on both sides of the Zhubaluo River and in the southeastern part near the boundary of the reserve. All of these regions have relatively high levels of human activity. However, there are more areas showing significant vegetation recovery than significant vegetation decline. The areas of significant and extremely significant increase of FVC were 286.24km^2^ and 498.44km^2^, accounting for 10.23% and 17.81% of the BNNR (see **Figure 6B**). The regions of vegetation restoration are predominantly situated around the snowy mountains in the north and in the southwestern part of the research region. In addition, in the eastern part of the BNNR, regions of vegetation recovery and deterioration are patchy.

#### 4.2.2 FVC change trend prediction

**Figure 7:**
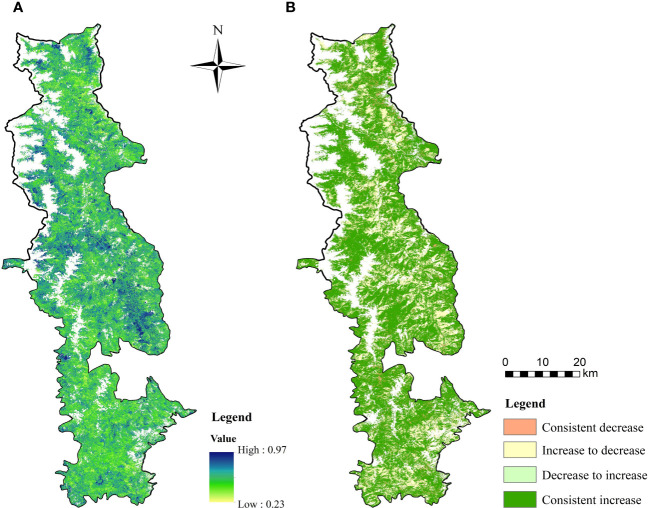
Projections of future trends in FVC in the BNNR combined the Hurst exponent with TS; **(A)** results of the Hurst exponent; **(B)** future change trend distribution of FVC.

The overall trend of vegetation change in the BNNR appears to be persistent, as indicated by the Hurst index research. Specifically, the area with a Hurst index greater than 0.5 accounted for 76.7% of the total area of the study area, while the percentage of the area less than 0.5 was 4.0% (see **Figure 7A**). The results of the TS analysis were integrated with the Hurst index to investigate the degree of sustainability of the FVC. The results of the research are depicted in **Figure 7B**), where the area transitioning from FVC reduction to growth and sustained growth are 24.17km^2^ and 1551.06km^2^, respectively. Conversely, the area transitioning from FVC improvement to degradation and sustained degradation are 82.71km^2^ and 487.13km^2^, respectively. Since the vegetation in most areas will continue the past trend, the distribution of FVC growth and decline in the future BNNR will basically coincide with the past trend.

### 4.3 FVC influence factor analysis

#### 4.3.1 Terrain effects of FVC

**Figure 8:**
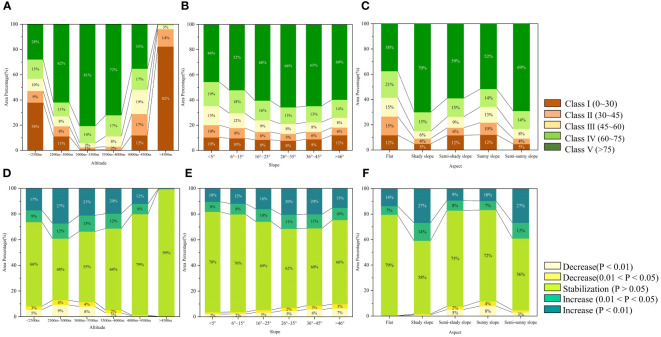
Proportion of each class FVC and FVC change trend in terrain factors; **(A)** proportion of FVC classes at various altitudes, **(B)** slopes, **(C)** and aspects; **(D)** proportion of FVC change trend at various altitudes, **(E)** slopes, **(F)** and aspects.

Based on the actual situation of the BNNR and related literature, its altitude, slope, and aspect were reclassified to explore the influence of topographic effect on FVC. The area proportion of FVC classes varies significantly with the elevation (see **Figure 8A**). The proportion of class I fluctuated greatly at different elevations. In the elevation range from 2500m to 3500m, the proportion of class V increases with the elevation, while the proportion of class I and class II decreases in this elevation range. However, in the area with elevation greater than 3500 m, the proportion of class I area increases with the elevation, especially in the area above 4500m, reaching up to 82%. But the proportion of class IV and class V in this area is even less than 1%. The variation trend of FVC also has an obvious terrain response at different elevations (see **Figure 8D**). Both vegetation deterioration (P< 0.05) and recovery (P< 0.05) are primarily located at altitudes intervals below 4500 m, and reach the maximum value between 2500 m and 3000 m. The proportion of regions with vegetation deterioration (P< 0.05) and recovery (P< 0.05) in FVC was 13% and 39%, respectively, at altitudes from 2500m to 3000m. It can be seen that the elevation zones with higher vegetation cover are also the places with the most drastic changes in vegetation cover. In addition, at elevations greater than 3000 m, the percentage of area with significant changes in vegetation decreases with elevation. Ultimately, at elevations greater than 4500m, vegetation is largely unchanged.

The proportion of area in class V increases with slope gradient from 46% for flat slopes to 66% for steep slopes when the slope is less than 35° (see **Figure 8B**). Concurrently, class IV, class III, and class II decreased by 6%, 7% and 5% respectively from flat slope to steep slope. The FVC significantly reduced proportion increases with the slope increasing, from 4% for flat slopes (<5°) to 10% for dangerous slopes (>46°) (see **Figure 8E**). The proportion of significant increase reaches its peak value in the slope range of 26°~45°. However, the difference in FVC between different slopes is relatively small compared to the difference in FVC between different altitudes and aspects.

The percentage of class V FVC in the BNNR is relatively higher on the shady slopes and semi-sunny slopes compared to other aspects (see **Figure 8C**). Additionally, the percentage of significant increases is higher for shady and semi-sunny slopes than for other slope orientations, while the percentage of significant decreases is relatively high for sunny and semi-sunny slopes (see **Figure 8F**). Specifically, shady slopes accounted for less than 2% of the area of significant FVC reductions, while sunny slopes accounted for 12% of the area of significant reductions. Conversely, the ratio of each grade of FVC does not differ much on the flats, and fewer areas with significant vegetative changes.

In summary, the distribution and changes in vegetative cover in the BNNR are likely more influenced by elevation compared to slope and aspect. Therefore, the vegetation in the BNNR with an altitude of 2500m to 3500m and located on the sunny slopes needs more attention.

#### 4.3.2 Climate effects on FVC

**Figure 9:**
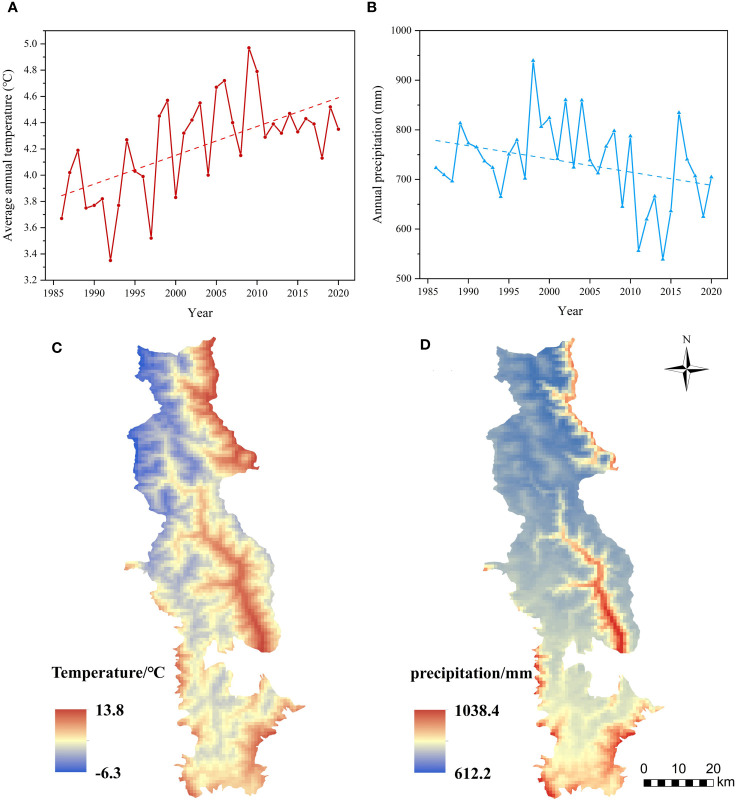
Temporal variation and distribution in space of temperature and precipitation; **(A)** temperature and **(B)** annual precipitation in the BNNR between 1986 and 2020; **(C)** differences in the space distribution of average temperature **(D)** precipitation.

The annual mean temperature of the BNNR stands at 4.2°C, displaying a fluctuating upward trend over the 35-year period. There’s a notable difference of 1.6°C between the highest (5.0°C in 2009) and lowest (3.4°C in 1992) annual mean temperatures (see **Figure 9**). Moreover, the average annual precipitation is recorded at 733.5 mm, with a slowly declining trend but significant inter-annual fluctuations. The disparity between the highest precipitation year (939.4 mm in 1998) and the lowest year (538.9 mm in 2014) is nearly 400 mm. Overall, temperature decreases from east to northwest in the BNNR, primarily influenced by topography. The lowest mean annual temperatures occur in the snow-covered mountainous areas at high elevations, while the highest mean annual temperatures are observed in the valleys at lower elevations. The spatial distribution of annual precipitation varies widely, with areas of higher precipitation distributed only in the southern part of the BNNR and along the banks of the Zhubaluo River.

**Figure 10:**
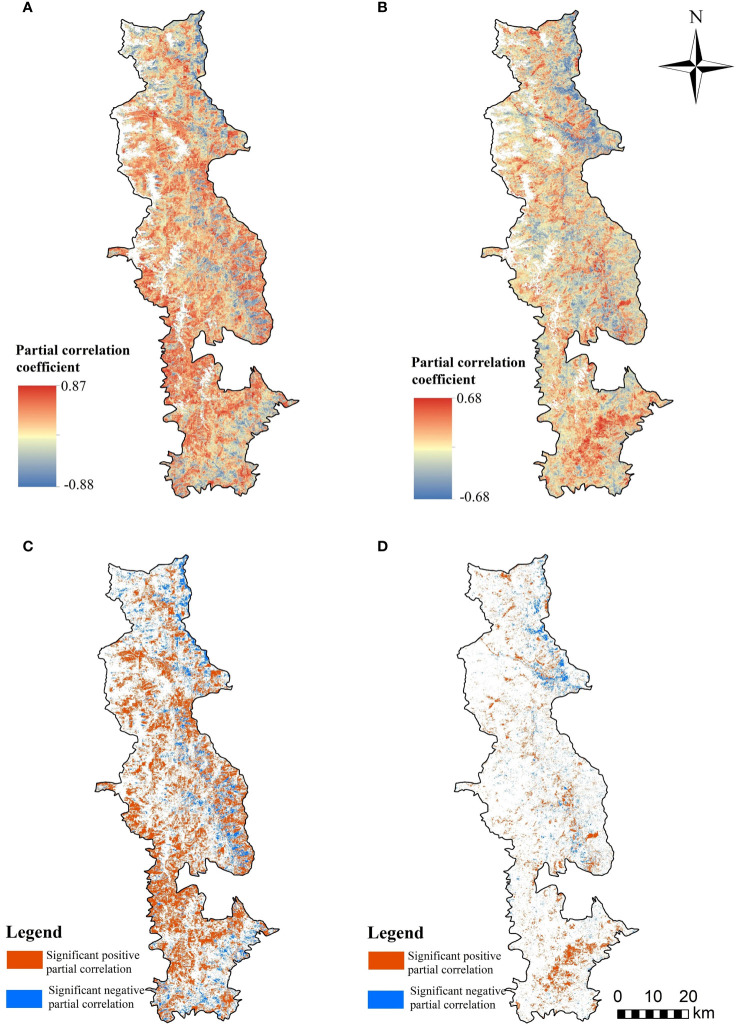
Partial correlation coefficient among FVC and climatic factors; **(A)** partial correlation among FVC and temperature **(B)** as well as precipitation; **(C)** significance test of partial correlation among FVC and annual mean temperature **(D)** as well as precipitation.

For the majority of the BNNR (62.65%), there was a positive correlation between FVC and temperature; of this, 27.90% of the region demonstrated a significant positive association (p<0.05) (see **Figures 10A, C**). The percentage of area where FVC is negatively correlated with annual mean temperature is 27.36%, with 7.63% of the negatively linked area is explained by a significant negative correlation. The southwestern and west-central sections of the BNNR are primarily home to the places where FVC and temperature have a major positive tie, while the negative correlation regions are primarily situated in the valley area of the BNNR’s northern part, on both sides of the Zhubaluo River in the central part, and in the southeastern part. Combined with the topography, it can be found that the negative correlation area is mostly distributed in the area of low elevation. The correlation between FVC and precipitation is indicated in **Figures 10B, D**), the area with positive correlation is 52.64% of the BNNR, while the negative correlation is 37.36%. Among them, significant positive correlation and significant negative correlation made up 7.79% and 3.11% of positive and negative correlations, respectively. The southern part of the BNNR is home to the majority of the regions where FVC and precipitation have a substantial positive association. The regions of negative correlation are located primarily in the northeastern portion. The findings revealed that the correlation between FVC and temperature is stronger in the BNNR compared to annual precipitation. The findings of the significance test confirm this view as well.

## 5 Discussion

### 5.1 FVC trend analysis

The lower FVC areas in the BNNR are concentrated in the snow-covered areas of the North and Midwest, as well as in the valleys. This finding is more in accordance with the local climate, the northwest part of the BNNR belongs to the Tibetan Plateau climate type, and the northeast part of the Jinsha River coast is owned by the arid river valley climate. These climatic influences render these areas more suitable for scrub and meadows, contributing to the lower FVC levels observed in the northern part of the BNNR (Chai et al., 2002). Conversely, regions around the towns of Xiaruo in the east-central part and Tacheng in the south, characterized by lower elevations and relatively higher moisture levels compared to the northern part, are more conducive to plant survival, resulting in higher vegetation cover.

Over the previous 35 years, there has been an overall upward trend in the BNNR’s average annual FVC. However, the growth rate has been relatively slow, at about 2.65% per decade. This finding is consistent with the results of other researches in the south-eastern part of the QTP and the Three Parallel Rivers region (Li et al., 2016; Wang et al., 2021b). This trend is primarily beneficial due to the active response of the local government in implementing major ecological protection projects such as the Natural Forest Protection Project, and the Yangtze River Basin Forest Protection Project and so on (Yang, 2017; Li et al., 2021; Deng et al., 2022b). These initiatives have led to a reduction in anthropogenic disturbances while promoting afforestation and vegetation recovery efforts in the BNNR. The spatial distribution results of FVC trends indicate that the recovery and decline of vegetation are patchily distributed along the banks of the Zhubaluo River. The low altitude of the area, the predominant vegetation category of cultivated vegetation, as well as the frequency of agricultural activities make the vegetation changes along the river banks relatively complex (Liu et al., 2014; Wang et al., 2021a).

The Hurst exponent prediction results indicate a continued upward trend in FVC within the BNNR. However, based on the spatial distribution of tendency projections, it can be seen that the ecosystems in the eastern and southern fringes of the protected areas are relatively fragile. These regions are still subject to pressure from vegetation degradation, influenced by both natural processes and human activities.

### 5.2 Topography and climate impact analysis on FVC

#### 5.2.1 Effect of topography on vegetation cover

The vertical distribution of vegetative cover in the BNNR shows that the region below 3300m is cool coniferous forest, warm coniferous forest and river valley scrub; from 3300m to 4000m is cool coniferous forest, and above 4000m is scrub meadow (Fan and Bai, 2021; Shi et al., 2021a). Different categories of vegetation are strongly affected by the scale of the terrain, and therefore trends also vary (Li et al., 2016). In the altitude range from 2500m to 4000m, the distribution of class IV and class V is predominant, and the sum of the two is more than 70%. And the percentage of significant increase in FVC was also relatively high in this elevation interval. The possible reason for this is that the soil here is dominated by mountain brown loam with high soil fertility. Therefore, this elevation range has a relatively high vegetation cover ability. Conversely, the relatively low-altitude areas below 2500m and above 4500m in the reserve have low vegetation cover, with grassland and scrub dominating the vegetation. The lower areas, below 2500 m, are characterized by dry, hot river valleys with minimal precipitation and high evaporation, making them unsuitable for forests but conducive to shrubs and grasses (Chai et al., 2002; Zhang et al., 2019). Areas above 4500 m are characterized by year-round snow and ice cover, slow accumulation of organic matter, and a shortened growing season for vegetation as the altitude rises (Li et al., 2016). Vegetation degradation is primarily observed at altitudes of 2500 m to 3500 m. According to the relevant data, the areas suitable for agriculture and animal husbandry in the study region are mainly located at low altitude (Wang et al., 2023). This suggests that the incidence of vegetation degradation in some low elevation regions may be related to anthropogenic factors.

The research results revealed that the FVC of the BNNR peaked at slopes ranging from 26° to 45°, with minimal differences in FVC among different slope categories. This may be related to the fact that gently sloping areas are subject to more frequent anthropogenic disturbances, but such disturbances are decreasing as the slope increases. Moreover, the percentage of area significantly reduced of FVC increases with slope, but the change is small. Slope has a relatively limited effect on the distribution and tendency of FVC, and the difference in FVC between different slopes in the BNNR is small. This phenomenon may be due to the fact that with the increase gradually of slope, soil water and nutrients will be more easily lost, so that the percentage of vegetation decline in the higher slope area will be slightly increased (Zhang et al., 2013). Relevant studies show that vegetative coverage in the TPRR is highest at a slope of 35° (Wang et al., 2021b), and have concluded that vegetation in this region is less affected by slope (Huang et al., 2023), which is in agreement with the findings of this research.

In the BNNR, vegetative cover is lower on sunny and semi-shady slopes compared to other slopes. Vegetation degradation predominantly takes place on sunny and semi-shady slopes. Revegetation occurs mainly on shady and semi- sunny slopes. This may be due to differences in radiation, temperature and water evapotranspiration on different slopes (Zhang et al., 2022a), leading to differences in vegetation distribution and growth. Combined with the fact that the main tree species in the BNNR are trees of the *Abies* and *Picea* (Shi et al., 2021a), which are better adapted to a cooler and colder climate, the shady slopes are more suitable for growth.

In summary, the variances and shifts in vegetative cover distribution within the BNNR are likely more influenced by elevation than by other factors.

#### 5.2.2 Impact of climate factors on vegetation cover

The QTP has long been acknowledged as a region experiencing more pronounced impacts from climate warming (Rangwala and Miller, 2012). Precipitation and temperature are the two primary climate elements that impact the growth of vegetative cover. In the BNNR, which is situated in the eastern section of the QTP, we found association between FVC and temperature was stronger than that of precipitation. This conclusion was further verified in the results of the significance test. Furthermore, this is in agreement with previous findings in the region that temperature is the leading element controlling or influencing vegetation cover increase in the region (Wang et al., 2021a; Guo et al., 2023). Owing to the relatively complicated terrain of the area, there are more pronounced spatial variations in the distribution of precipitation and temperature (Zhang et al., 2018; Guo et al., 2023), resulting in different correlations between FVC and climate elements in different areas. The relationship between temperature and elevation elements tends to have a more pronounced linear negative correlation, leading to possible differences in the response of vegetation dynamics to temperature. Wang et al. on the Tibetan Plateau showed that the correlation between vegetation and temperature varied at different altitudes, especially at altitudes above 2400 m, where temperature, instead of precipitation, played a major role in regulating the vegetation ecosystem (Wang et al., 2020).

Additionally, to some degree, the rise in temperature might encourage the development of plants (Wei et al., 2022). Several studies have shown that in the summer, temperature and tree radial growth are favorably connected (Kang et al., 2021). The growth of dominant tree species such as *Abies george* and *Picea asperata* in the BNNR was positively correlated with temperature (Shi et al., 2021a). This may also be one of the reasons for the relatively strong correlation between temperature and vegetation cover. However, it has also been suggested that raised temperature would lead to an increase in plant respiration rate (Hua et al., 2019). Rising temperatures will accelerate snowmelt, potentially yielding diverse effects on vegetation. On the one hand, early snowmelt and longer growing seasons will increase vegetation productivity, but on the other hand, early snowmelt may also lead to low temperatures in the early spring and reduced snowmelt in the summer leading to droughts and a decrease in vegetation productivity, which is mainly related to seasonal and geographic variations (Wang et al., 2013). Vegetation phenology is closely related to climate. It has been suggested that climate warming leads to a longer growing season for vegetation, which promotes plant growth and biomass accumulation (Wheeler et al., 2017). As there is a strong link between vegetation cover and biomass (Qin et al., 2022). Therefore, changes in vegetation phenology as a result of climate warming have had a relatively positive effect on vegetation cover.

Regarding the regional distribution of positive correlations with precipitation, the southern part of the BNNR exhibits a vegetation cover more susceptible to precipitation, possibly linked to variations in the distribution of vegetation categories. In the past, temperature has been more influential on vegetation than rainfall. However, as temperature continues to rise and annual precipitation tends to reduce, the association of vegetative growth with precipitation may become stronger in the future. In the context of global warming, the frequency and magnitude of climate extremes have increased. Hazards such as droughts, temperature extremes, and floods can cause varying degrees of vegetation degradation (Manoranjan et al., 2024). Therefore, the mechanism of vegetation response to global warming in high-altitude mountainous areas needs to be further investigated.

### 5.3 Limitations and deficiencies

This study focuses on analyzing the long time-series variation patterns of vegetation. Vegetation phenology is also a key indicator for evaluating climate impacts on vegetation, carbon cycling, and interannual changes in ecosystem productivity (Wu et al., 2021; Fu et al., 2022a). This requires high-quality images of the growing season and data on the distribution of vegetation types. However, Landsat imagery capturing the vegetation growing season in the study area is significantly impacted by clouds and cloud shadows. This challenge may necessitate the integration of other imagery sources to generate higher-quality growing season imagery. Additionally, there is currently a temporary absence of vegetation type data in the study area. This gap could be addressed in the future with the generation of spatial distribution data on vegetation types in protected areas using more advanced equipment and methods.

## 6 Conclusion

The spatial and temporal variations, as well as the developmental tendencies of FVC, were investigated using the TS and MK methods, while the prediction of FVC was analyzed through the Hurst exponent. Topographic effects of FVC and its trends were also analyzed. Furthermore, to examine the correlation between FVC and climatic elements at the pixel level, a partial correlation coefficient was applied. This research primarily yielded the following key findings: FVC of the BNNR exhibited an upward trend from 1986 to 2020, with the mean FVC value growing from 59.40% in 1986 to 68.67% in 2020. The proportion of class I, II, III and IV decreased, while the proportion of class V FVC increased significantly. The spatially distributed differences of FVC in the BNNR are very obvious. Higher FVC regions are mostly situated at the east-central and southern portions of the BNNR. Snow-covered regions and valleys in the northern and west-central portions of the BNNR have lower FVC. FVC trends in the BNNR reveal two distinct patterns: a declining trend that accounted for 22.13% (a significant decrease of 6.42%) and a growing trend that accounted for 59.03% (a significant rise of 28.04%) of the area, respectively. Hurst exponent analysis indicated that most regions in the BNNR will continue to have an increased trend of FVC in the future. Topographic factors significantly influence the shift in FVC trends and spatial distribution. The vegetation coverage is higher in the height range from 2500m to 4000m, and the percentage of significant increase in FVC is also higher in this region. The area of significant vegetation change decreases with elevation. Slope has a limited effect on FVC distribution and trend, with minimal differences observed between different slopes. FVC tends to be lower on sunny and semi-shady slopes compared to other slopes, with vegetation degradation predominantly occurring in these areas. The following are the primary ways that climate variables affect the vegetation in this area: the FVC of the BNNR was positively correlated with precipitation and temperature, making up 52.64% and 62.65% of the total area, correspondingly. The temperature factor has a relatively strong ability to influence the vegetation cover with high spatial correlation.

## Data availability statement

The original contributions presented in the study are included in the article/supplementary material. Further inquiries can be directed to the corresponding author.

## Author contributions

ZX: Formal Analysis, Investigation, Methodology, Software, Visualization, Writing – review & editing, Validation, Writing – original draft. XS: Formal Analysis, Investigation, Methodology, Software, Visualization, Writing – review & editing. SG: Investigation, Resources, Validation, Writing – review & editing. QS: Investigation, Resources, Validation, Writing – review & editing. YY: Investigation, Resources, Validation, Writing – review & editing. LC: Conceptualization, Data curation, Funding acquisition, Investigation, Resources, Supervision, Validation, Writing – review & editing.
